# Loss of alanine-glyoxylate and serine-pyruvate aminotransferase expression accelerated the progression of hepatocellular carcinoma and predicted poor prognosis

**DOI:** 10.1186/s12967-019-02138-5

**Published:** 2019-11-26

**Authors:** Yufeng Sun, Wenchao Li, Shiqi Shen, Xuejing Yang, Bing Lu, Xiaojing Zhang, Peng Lu, Yi Shen, Juling Ji

**Affiliations:** 1 Department of Pathology, Medical School of Nantong University, Nantong, China; 2 Basic Medical Research Centre in Medical College of Nantong University, Nantong, China; 3 Department of Epidemiology and Medical Statistics, Public Health School of Nantong University, Nantong, China; 4 Medical School of Nantong University, Nantong, China; 5 Department of Clinical Biobank, Affiliated Hospital of Nantong University, Nantong, China; 6 Department of Biostatistics, Public Health School of Shandong University, Jinan, China

**Keywords:** Hepatocellular carcinoma, Systematic review, Alanine-glyoxylate and serine-pyruvate aminotransferase, Diagnostic marker, Prognostic marker

## Abstract

**Background:**

Accumulated studies reported abnormal gene expression profiles of hepatocellular carcinoma (HCC) by cDNA microarray. We tried to merge cDNA microarray data from different studies to search for stably changed genes, and to find out better diagnostic and prognostic markers for HCC.

**Methods:**

A systematic review was performed by searching publications indexed in Pubmed from March 1, 2001 to July 1, 2016. Studies that reporting cDNA microarray profiles in HCC, containing both tumor and nontumor data and published in English-language were retrieved. The differentially expressed genes from eligible studies were summarized and ranked according to the frequency. High frequency genes were subjected to survival analyses. The expression and prognostic value of alanine-glyoxylate and serine-pyruvate aminotransferase (AGXT) was further evaluated in HCC datasets in Oncomine and an independent HCC tissue array cohort. The role of AGXT in HCC progression was evaluated by proliferation and migration assays in a human HCC cell line.

**Results:**

A total of 43 eligible studies that containing 1917 HCC patients were included, a list of 2022 non redundant abnormally expressed genes in HCC were extracted. The frequencies of reported genes were ranked. We finally obtained a list of only five genes (AGXT; ALDOB; CYP2E1; IGFBP3; TOP2A) that were differentially expressed in tumor and nontumor tissues across studies and were significantly correlated to HCC prognosis. Only AGXT had not been reported in HCC. Reduced expression of AGXT reflected poor differentiation of HCC and predicts poor survival. Knocking down of AGXT enhanced cell proliferation and migration of HCC cell line.

**Conclusions:**

The present study supported the feasibility and necessity of systematic review on discovering new and reliable biomarkers for HCC. We also identified a list of high frequency prognostic genes and emphasized a critical role of AGXT deletion during HCC progression.

## Background

Liver cancer is the third leading cause of cancer death and the sixth most common cancer worldwide [1]. Hepatocellular carcinoma (HCC) accounts for 70–85% of the total liver cancer burden. The accurate diagnosis and proper treatment of HCC is particularly challenging. Options for HCC treatment remain limited, surgical resection is considered the only “curative treatment”. But HCC patients do not have overt symptoms in the early stages, 80% of patients have widespread HCC at the time of diagnosis and are not candidates for surgical treatment. Even with surgical resection, the 5-year survival rate is poor (about 38%) [2].

Accumulated data from high-throughput analyses by cDNA microarray provide an accurate landscape of gene expression in HCC, and revealed a lot of pathogenic and prognostic genes for HCCs, thus enabled us to delineate some of the key events that might dominate tumor development and progression. Hopefully, translation of this knowledge into new targets and biomarkers might impact HCC decision making, and ultimately improve patient’s outcomes [3]. However, these studies were performed with different platforms and in different populations, the suggested diagnostic markers and potential therapeutic targets for HCC varied across studies, hence prevented the application of these findings. It’s the high time to merge these cDNA microarray data from different studies using different platforms, to search those widely and stably changed genes, and to find out better diagnostic markers and potential therapeutic targets for HCC.

In the present study, we performed a systematic review of studies that reported cDNA microarray data for both tumor and nontumor liver tissues of HCC patients and came up with a list of five genes that were differentially expressed in tumor and nontumor tissues across different studies and were significantly correlated to HCC prognosis. Among the five genes, except for alanine-glyoxylate and serine-pyruvate aminotransferase (AGXT) which is an essential gene involved in glyoxylate detoxification, the other four genes were well documented in HCC. Here, we reported that AGXT was involved in the progression of HCC and loss of AGXT expression predicted poor prognosis of HCC. AGXT might be a novel diagnostic and prognostic marker and a potential therapeutic target for HCC.

## Methods

### Data sources and search strategy

Literature retrieval was originally performed with PubMed, a free search engine accessing primarily the MEDLINE database of references and abstracts on life sciences and biomedical topics. The relevant Mesh Terms were chose as the key words, including hepatocellular carcinoma, liver neoplasms, gene expression profiling, human, gene expression regulation, oligonucleotide array sequence analysis. Date of publications was restricted to July 2016. Besides, the literature must be published in English language. In addition to use (((((((carcinoma, hepatocellular[MeSH Terms]) AND liver neoplasms[MeSH Terms]) AND gene expression profiling[MeSH Terms]) AND human[MeSH Terms]) AND gene expression regulation[MeSH Terms]) AND oligonucleotide array sequence analysis[MeSH Terms])) AND (“1781/01/01”[Date-MeSH]: “2016/06/30”[Date-MeSH]) as the search strategy to obtain literatures, we also reviewed the bibliographies of eligible studies as well as those of relevant review articles to identify additional studies not captured by our database searches. To identify all potentially eligible studies, two investigators independently conducted structured searches in selected databases.

### Study inclusion criteria

Eligible studies were included in the systematic review if they met the following criteria: (1) they were gene expression studies in hepatocellular carcinoma; (2) they used tissue samples obtained from surgically resected tumor and corresponding non-tumor or normal tissues in human for comparison; (3) validation of method and sample set were reported; (4) clinical and experimental study. Articles were excluded based on the following criteria: (1) review articles or letters; (2) non-liver cancer; (3) non-gene expression profile; (4) non-human tissue samples.

### Data extraction

From the full text and corresponding additional information, the following items were eligible to collect and record for each study: authors, year of publication, region, selection number and characteristics of recruited liver cancer patients, the members of abnormally expressed genes in liver cancer. Two investigators (K. D. and YL. J.) independently evaluated and extracted the data with the inclusion criteria. Conflicts in study selection at this stage were resolved by consensus, referring back to the original article in consultation with the principal.

### Gene statistics and screening

For genes that have multiple names/synonyms, we standardized the gene name for later work by using widely accepted and used HGNC database (https://www.genenames.org/). Frequency and composition analysis of the abnormally expressed genes in hepatocellular carcinomas were conducted by IBM SPSS Statistics 20.0 software (Endicott, New York, NY). The genes presented in more than four studies were regarded as high frequent genes. The expression of the high frequent genes in other malignant tumors were obtained from Oncomine (https://www.oncomine.org/). To identify a prognostic gene list across different studies, the prognostic value of these high frequent genes were evaluated by survival risk prediction in a previously described cohort of 247 Chinese HCC patients with publicly available Affymetrix U133A array data (National Center for Biotechnology Information [NCBI] Gene Expression Omnibus [GEO] Accession number GSE14520) [4]. BRB-Array Tools (version 4.3.1) was used for survival risk prediction. By reviewing related publications, the prognostic genes that have been intensively investigated were excluded, and the only one gene AGXT (alanine-glyoxylate and serine-pyruvate aminotransferase) that had not been reported in HCC were chosen for further validation.

### Tissue microarray and immunohistochemistry staining

Tissue microarrays of 192 HCC patients were used for validation. All of the patients underwent curative hepatectomy for primary HCC at the Affiliated Hospital of Nantong University between March 2004 and August 2009. No patients received either radiotherapy or chemotherapy before the surgery. The study was performed on the basis of the protocol approved by the Declaration of Helsinki, and written informed consent was obtained from all patients. The histological Grading of HCC was defined according to the Edmondson–Steiner grading system. In the present study, grade I and II were termed as well differentiated, grade III was termed as moderate differentiated, and grade IV was termed as poor differentiated. Tumor stage was assigned according to the American Joint Committee on Cancer TNM staging. Patients were followed up every 2–3 months during the 1st year after surgery and every 3–6 months thereafter until September 2016. Totally 101 out of the 192 HCC patients had integrated clinical and follow up data (Additional file 1: Table S1).

Immunohistochemistry was performed with Envision + kits (DAKO, Carpinteria, CA) according to the manufacturer’s instructions [4], of which positive staining appeared in brown. The primary antibodies used were rabbit anti-AGXT monoclonal antibody (1:100, Abcam). Immunostained slides were analyzed with a semi quantitative scoring approach which combined staining intensity and percentage of positive cells: grade 0 for no reaction or focal weak reaction; grade 1 for intense focal or diffuse weak reaction; grade 2 for moderate diffuse reaction; and grade 3 for intense diffuse reaction, and the corresponding slides were scored from 0 to 3 respectively. The staining scores were evaluated independently by two pathologists who were blinded to the clinical outcomes.

### Cell culture

Human HCC cell lines were cultured in Dulbecco’s Modified Eagle’s Medium (DMEM) (Gibco, CA, USA) supplemented with 10% fetal bovine serum (FBS, Gibco, Carlsbad, USA) in a 5% CO_2_ atmosphere at 37 °C.

### siRNA transfection

Huh-7 cells were seeded into 6-well culture plates and transfected with small interfering RNAs (siRNAs) against human AGXT (siAGXT) (SASI_Hs01_00153951, SASI_Hs01_00153952 and SASI_Hs01_00153953, Sigma) using Lipofectamine™ 2000 (Invitrogen, Carlsbad, CA), at a final concentration of 75 nm. Non-targeting control siRNA (siNC, Sigma) was used as negative control. Cy3 labeled siRNA transfection control (Cy3-siTC, RiboBio, Guangzhou, China) was used to optimize siRNA concentration for transfection. Knockdown efficiency was determined by real-time quantitative reverse transcription-PCR (RT-PCR) 48 h after the transfection.

### Real-time quantitative RT-PCR

The RNAs from Huh-7 or HepG2 cells were reverse-transcribed with Thermoscript RT-PCR system (Invitrogen). Real-time quantitative PCR was performed on RotorGene 3000 instrument (Corbett Research, New South Wales, Australia) with SYBR qPCR master lit (TaKaRa, Dalian, China). Gene specific primers were: AGXT forward primer 5′-CTGGGGACTCCTTCCTGGTT-3′, AGXT reverses primer 5′ -CACCTCCTGCAGTGTGTAGT-3′; β-actin forward primer 5′-TTGTTACAGGAAGTCCCTTGCC-3′, β-actin reverses primer 5′ -ATGCTATCACCTCCCCTGTGTG-3′. Relative gene expression was normalized to housekeeping gene β-actin and calculated as 2 − ΔCτ.

### Western blot

For western blot analysis, whole cell protein was extracted by RIPA lysis buffer (Beyotime, Shanghai) according to the manufacturer’s instructions. Equal amounts of protein (20 µg) were separated on 10% SDS PAGE gel and transferred onto polyvinyldifluoride (PVDF) membranes (Millipore). PVDF membranes were blocked with 5% non-fat milk for 1 h, then incubated with specific primary antibodies for anti-AGXT antibody (Abcam, Cambridge, UK), and β-actin (CST, Boston, America) at 4 °C overnight, then incubated with horseradish peroxidase-conjugated secondary antibody for an additional 1 h at room temperature. The protein expression was visualized with the ECL chemiluminescence detection system (Biorad).

### Proliferation and migration assays

Proliferation ability of HCC cells was measured by cell counting using a Counting Chamber or Cell Counting Kit-8 reagent (CCK8, Dojindo Laboratories, Kumamoto, Japan) according to the manufacturer’s recommendation. Cells were seeded at 1 × 10^5^ cells per 25 cm^2^ Corning cell culture flask or 2 × 10^3^ cells per well in 96-well plates and cultured for 0 h, 24 h, 48 h, 72 h, 96 h, 120 h and 144 h. For cells cultured in 96-well plates, CCK8 solution was added (10 μl each well) and incubated at 37 °C for 2 h. The optical density readings at 450 nm were determined by a micro plate reader (Bio-Rad, Tokyo, Japan).

For transwell migration assays, a single cell suspension of 50,000 Huh-7 and HepG2 cells, or siAGXT/siNC treated Huh-7 cells resuspended in 200 μl serum-free DMEM were plated in the upper chambers (Millicell, 8.0 μm; Corning, USA), 750 μl DMEM medium with 10% FBS was used as a chemoattractant in the lower chambers. After 24 h, nonmigrating cells were removed from the upper surface softly by a cotton swab. The cells that migrated through the membrane to the lower surface were fixed with 4% paraformaldehyde and stained with 0.5% crystal violet, then counted under a microscope (Olympus) at 200-fold magnification, five fields were counted for each well.

### Cell cycle assay and apoptosis assay

HCC cells were seeded at 2 × 10^5^ per well in 6 well plates. After overnight incubation, cells were treated with siAGXT or siNC for 72 h. After treatment, HCC cells were digested using 0.25% pancreatic enzyme, washed twice with PBS, fixed in precooled 70% cold ethanol at 4 °C for 24 h. Cells were centrifuged again, washed with cold PBS twice, and stained with Propidium iodide (0.1 mg/ml) (Propidium iodide, PI; Beyotime) at 37 °C in the dark for 30 min. DNA contents were measured with a BD FACS Calibur system (BD Biosciences, Franklin Lake, NJ). Data were analyzed using ModFit LTTM software (Verity Software House, Topsham, ME). For apoptosis assay, cells were harvested, washed, and resuspended with PBS and stained with BD Annexin V-FITC Apoptosis Detection Kit. Data was acquired using a BD FACS Calibur system and BD FACSuite software (BD Biosciences).

### Statistical analysis

Statistical analyses were performed with Graphpad Prism 6.0 (Graphpad Software, Inc., La Jolla, CA) or IBM SPSS Statistics 20.0 software (Endicott, New York, NY). Quantitative data were expressed as mean ± SD. Comparisons between groups were made by Student’s t-test or two-way ANOVA. Categorical data were evaluated by the χ^2^ test. All P-values were two-sided and the statistical significance was defined as P < 0.05. All experiments were performed in triplicates.

## Results

### Study selection and characteristics of included studies

**Fig. 1:**
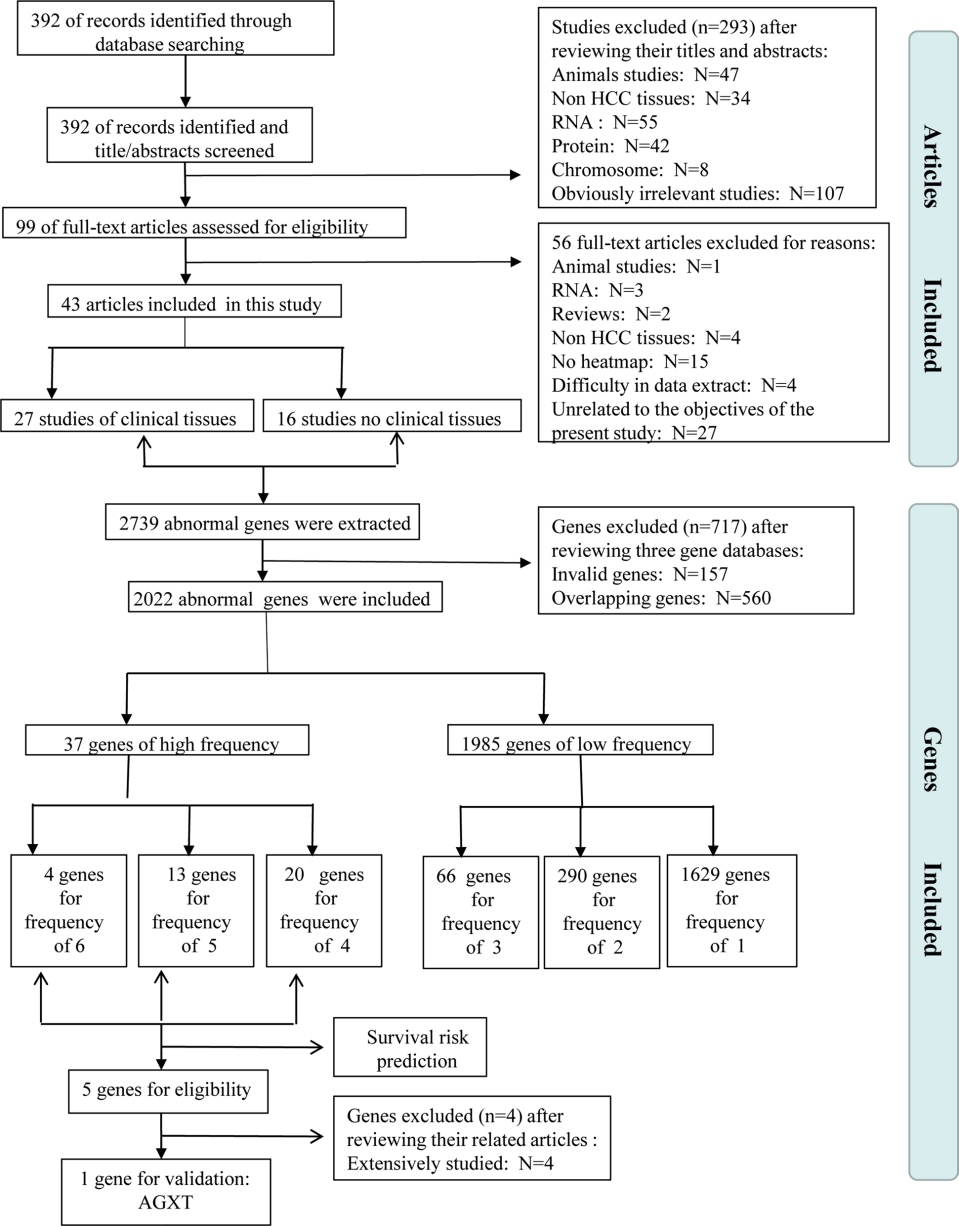
Flowchart of study selection and gene screening

According to the literature retrieval strategy, 392 studies were identified in the initial search (Fig. 1). Based on the independent screening of titles and abstracts by two investigators (K. D. and YL. J.), 293 studies were excluded, of which 47 were animal studies, 34 were not performed with liver cancer tissues, 105 were not gene expression profiling studies, 107 articles were not correlated with the present study, and 99 papers remained. The full text of these remaining 99 studies was retrieved and further assessed for eligibility. Of these 99 publications, 56 were excluded (one was animal study, 3 were small RNA studies, 2 were review articles, 4 were non-HCC, 15 were excluded because they did not have gene expression profiling data, 4 studies had difficult in data extraction, 27 were excluded because they were irrelevant to our study). In total, 43 articles were included. All of these 43 articles were based on HCC cohort study containing a total of 1917 HCC patients. After a preliminary check of these 43 articles, we included “Author”, “Year of publication”, “Country”, “Number of patients” (NP), “Number of significant genes” (NG),”Clinical tissue” (Tissue), “Contain adjacent tissue” (C&A), “Hepatitis B surface antigen” (HBs-Ag), “Hepatitis C antibody” (HCV-Ab), “Alcoholism “, “Tumor size” (TS),”Tumor differentiations” (TD), and “AFP” as the baseline variables to characterize the enrolled studies. Thirteen studies contained the information of both hepatitis B surface antigen (HBs-Ag) and hepatitis C antibody (HCV-Ab), seven studies only enrolled person carrying HCV-Ab, four studies only included patients carrying HBs-Ag, and two studies had the information of alcoholism. So we combined these three variables into the variable “Etiology”. Only six articles provided the record of tumor size, of which four articles [5–8] used 5 cm and two articles [9, 10] used 3 cm to distinguish large or small tumor. The differentiation of tumors was described as highly, moderately and poorly differentiated in four articles [5, 11–13]. Few literatures discussed AFP. Due to the excessive missing information for tumor size, tumor differentiation and AFP, only “Author”, “Year of publication”, “Country”, “Number of patients” (NP), “Number of significant genes” (NG),”Clinical tissue” (Tissue), “Contain adjacent tissue” (C&A) and “Etiology” were included. These main characteristics of the 43 studies were summarized in Table 1.

**Table 1:** Characteristics of the 43 included studies

Article	Year of publication	Country	NP	NG	Tissue	C&A	Etiology
Engelmann [14]	2015	Germany	15	30	F	T	
Itzel [15]	2015	USA	163	61	F	F	
Yu [16]	2015	China	154	67	T	F	HBV
Shi [17]	2014	China and Singapore	26	6	T	F	
Mayinuer [18]	2013	Japan	152	154	T	F	
Gehrau [19]	2012	USA	107	17	F	F	HCV
Nagai [11]	2012	Japan	21	25	T	F	HCV
Roessler [20]	2012	China	76	6	F	F	HBV
Sato [21]	2012	Japan	48	20	T	F	HCV
Yu [22]	2012	China	100	59	T	F	HBV
Zekri [23]	2012	Egypt	31	25	T	T	HCV
Satow [24]	2010	Japan	84	124	T	T	
De Giorgi [25]	2009	Italy	21	39	F	F	HCV
Sugawara [26]	2010	Japan	1CL	36	F	F	
Derambure [12]	2008	France	40	63	T	F	HBV, HCV, alcoholism
Gao [9]	2008	Japan	38	24	T	T	HCV, HBV
Guo [27]	2008	China	3CL	17	F	F	
Hamaguchi [10]	2008	Japan	60	22	T	F	
Kittaka [5]	2008	Japan	100	25	T	F	HCV, HBV
Sakai [13]	2008	Japan	12	93	F	T	HCV
Skawran [28]	2008	Germany	32	185	F	F	HBV, HCV
Yamashita [29]	2008	China	40	71	T	F	HBV
Lin [30]	2007	Taiwan	–	19	F	F	
Liu [31]	2007	China	1	45	T	F	
Wang [32]	2007	Singapore	80	57	T	F	HCV, HBV
Wang [33]	2007	USA	14	134	F	F	
Danenberg [34]	2006	USA	1CL	50	F	F	
Chen [35]	2005	USA	63	54	F	F	
Ge [36]	2005	Japan	36	58	F	F	
Iizuka [37]	2005	Japan	76	110	T	F	HCV, HBV
Matoba [38]	2005	Japan	33	46	T	F	HCV, HBV
Nam [6]	2005	Korea	42	92	T	F	
Wong [39]	2005	China	10	55	F	F	HBV, HCV
Breuhahn [40]	2004	Germany	39	118	T	T	HBV, HCV, alcoholism
Chen [41]	2004	USA	–	29	T	F	
Lee [7]	2004	Korea	10	20	T	F	HCV, HBV
Kurokawa [42]	2004	Japan	20	63	T	F	HBV, HCV
Smith [8]	2003	USA	20	50	T	F	HCV
Kim [43]	2003	USA	74	30	T	F	
Chung [44]	2002	Korea	8	62	T	T	
Iizuka [45]	2002	Japan	51	83	T	F	HBV, HCV
Lee [46]	2002	USA	19CL	119	F	F	
Okabe [47]	2001	Japan	20	–	T	T	HBV, HCV

*NP* number of patients, *NG* number of significant genes, *Tissue* clinical tissue, *C&A* contain adjacent tissue, *CL* cell line, *T* true, *F* false

### Abnormally expressed genes in HCC across different studies

**Fig. 2:**
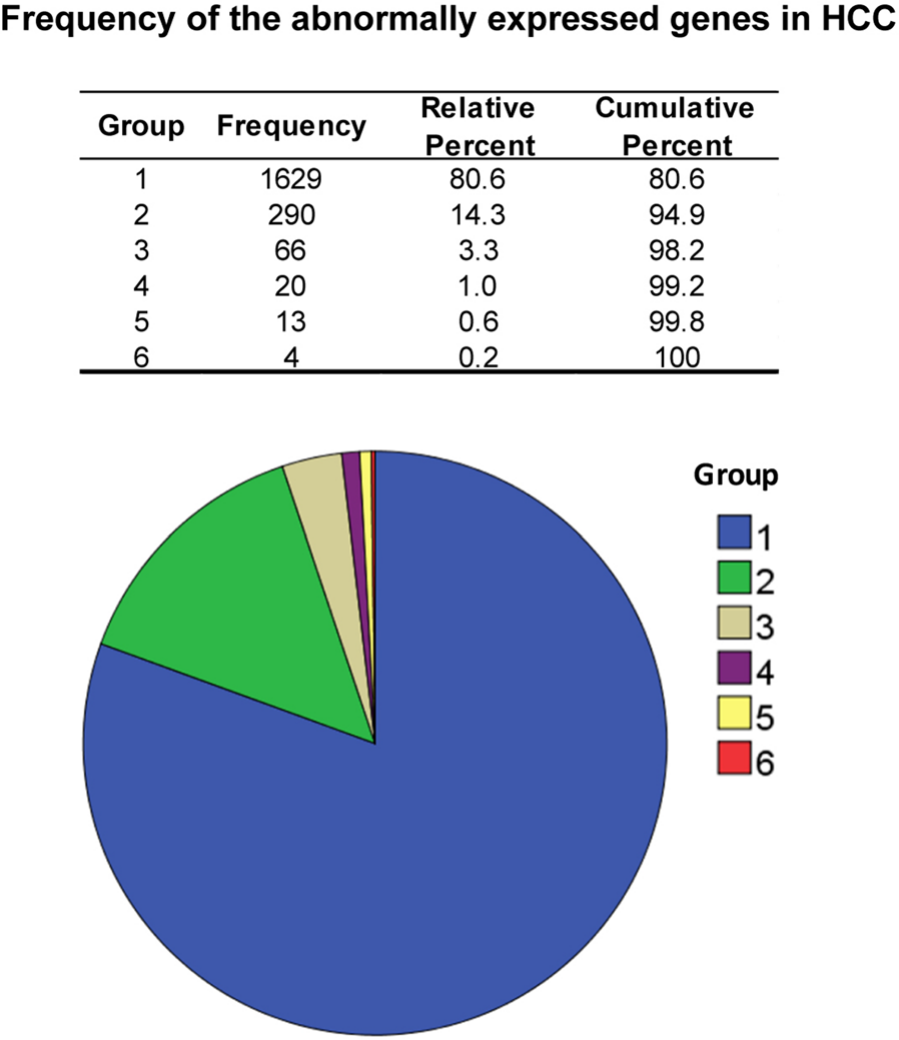
Frequency analysis of the abnormally expressed genes in hepatocellular carcinoma. Data presented in the frequency table and pie chart were from the 43 studies included

Totally 2739 abnormally expressed genes in HCC were extracted from the 43 studies. After standardization of gene names according to HGNC database (https://www.genenames.org/), 2576 genes remained (including repetitive). When repetitive genes were excluded by SPSS 20.0, 2022 non-redundant genes remained (Fig. 1). The frequency of each gene that appeared in the 43 studies included was counted. The frequency ranged from 1 to 6, with 6 the highest frequency, while 1 the lowest frequency (Fig. 2, Additional file 2: Table S2). Proportion of frequency 6 was the minimum, the frequency 1 constituted the largest proportion of all. Genes that appeared ≥ 4 times were regarded as genes of high frequency genes (37 genes), and genes that appeared ≤ 3 times were regarded as genes of low frequency (1985 genes). There are four genes that appeared in six studies (0.2%), including C8A, MT1E, MT1H and NNMT; 13 genes appeared in five studies (0.6%), including: AFP, ALDOB, C9, CXCL12, CYP2E1, HPX, IGF2, IGFBP3, MT1F, MT2A, RAF1, SULT1E1 and TOP2A; 20 genes appeared in four studies (1.0%), including: AGXT, AKR1B10, BCL2, C4A, C6, CES1, CLU, CYP2E1, EPCAM, FCN3, FDPS, FOS, GPC3, MFSD2A, PDCD4, PPIB, RGS5, SAA1, SAA4 and TAT; 66 genes appeared in three studies (3.3%); 290 genes appeared in two studies (14.3%); 1629 genes only appeared once (80.6%).

The publication history of these 37 high frequent genes in HCC was searched in Pubmed (Additional file 3: Table S3). About half of these genes have been extensively studied in HCC and appeared in more than 5 directly related studies, including: AFP on the top with 824 publications, followed by EPCAM, GPC3, CYP2E1, FOS, BCL2, IGF2, CXCL12, AKR1B10, PDCD4, TAT, RAF1, IGFBP3 and TOP2A, while seven genes has not been reported in HCC by the time of literature searching, including: AGXT, FCN3, FDPS, MFSD2A, RGS5, SAA1 and SAA4. The relative expression of these 37 high frequent genes in liver cancer and other malignant tumors were obtained from Oncomine (https://www.oncomine.org/) and provided in Additional file 3: Table S3.

### Genes significantly associated with the survival of HCC patients

**Fig. 3:**
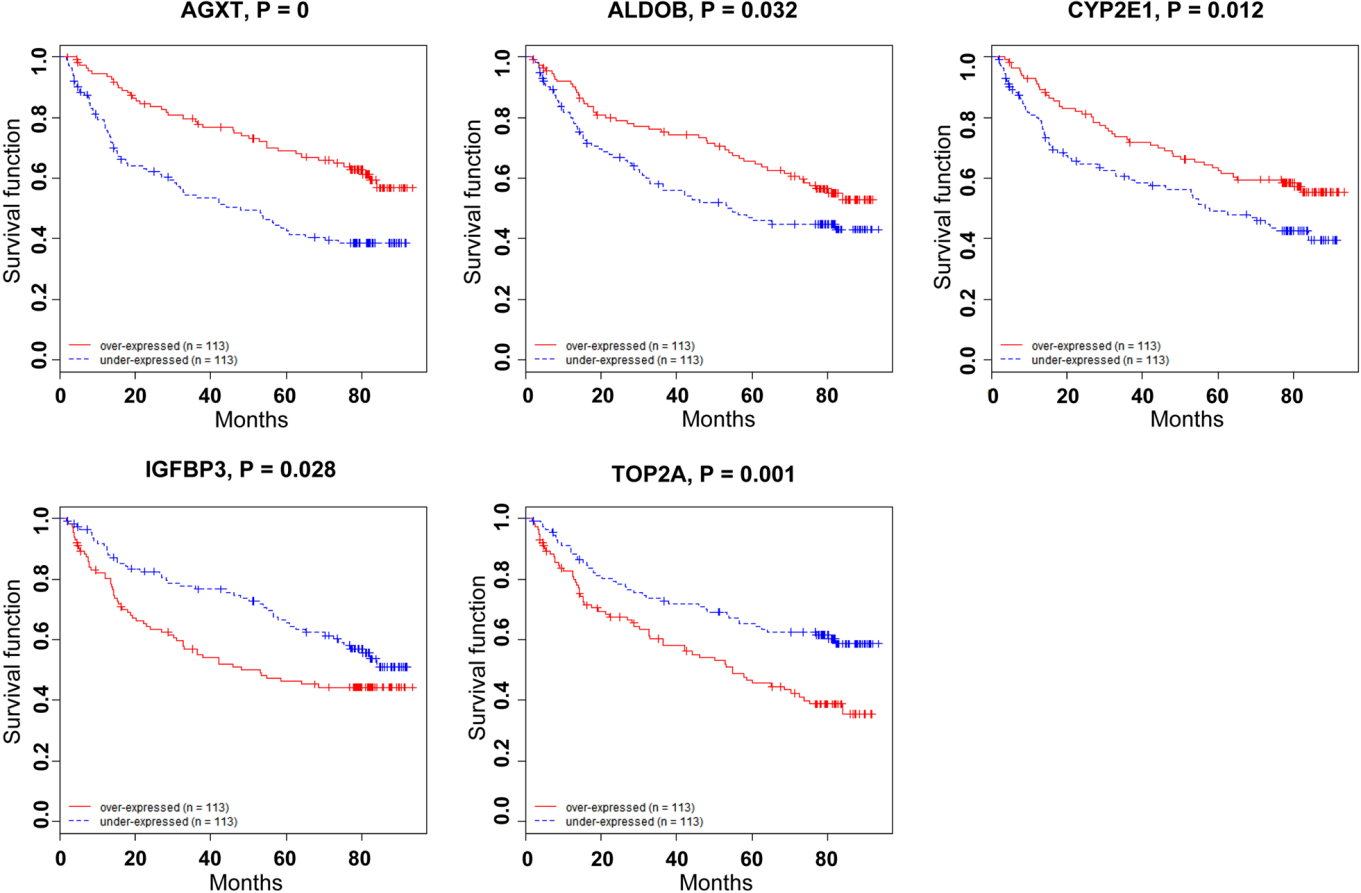
The expression of AGXT, ALDOB, CYP2E1, IGFBP3 and TOP2A predicted clinical outcome in HCC. The prognostic value of the 37 high frequent genes was evaluated by survival risk prediction in a cohort of 247 Chinese HCC patients (Gene Expression Omnibus Accession number GSE14520). BRB-Array Tools (version 4.3.1) was used for survival risk prediction. Among the 37 genes, under-expression of three genes (alanine-glyoxylate and serine-pyruvate aminotransferase, AGXT; aldolase B, ALDOB; cytochrome P450 family 2 subfamily E member 1, CYP2E1) and over-expression of two genes (insulin-like growth factor binding protein-3, IGFBP3; topoisomerase 2α, TOP2A) were significantly correlated to poor prognosis. Red line represented samples with over-expressed gene, and blue line represented samples with under-expressed gene

The prognostic value of the 37 high frequent genes were evaluated by survival risk prediction in a previously described cohort of 247 Chinese HCC patients with publicly available Affymetrix U133A array data (Gene Expression Omnibus accession number GSE14520) [4]. BRB-Array Tools (version 4.3.1) was used for survival risk prediction. Among the 37 genes, under-expression of three genes (alanine-glyoxylate and serine-pyruvate aminotransferase, AGXT; aldolase B, ALDOB; cytochrome P450 family 2 subfamily E member 1, CYP2E1) and over-expression of two genes (insulin-like growth factor binding protein-3, IGFBP3; topoisomerase 2α, TOP2A) were significantly correlated to poor prognosis (Fig. 3). By literature reviewing, among the five genes, four have been well documented in HCC, including: ALDOB [48–50], IGFBP3 [51–53], CYP2E1 [54–56] and TOP2A [57, 58], while AGXT was mostly studies in primary hyperoxaluria type 1 [59, 60], there are little reports of AGXT in HCC. Thus, AGXT was chosen for further validation.

### AGXT was highly expressed in liver but lost in HCC

**Fig. 4:**
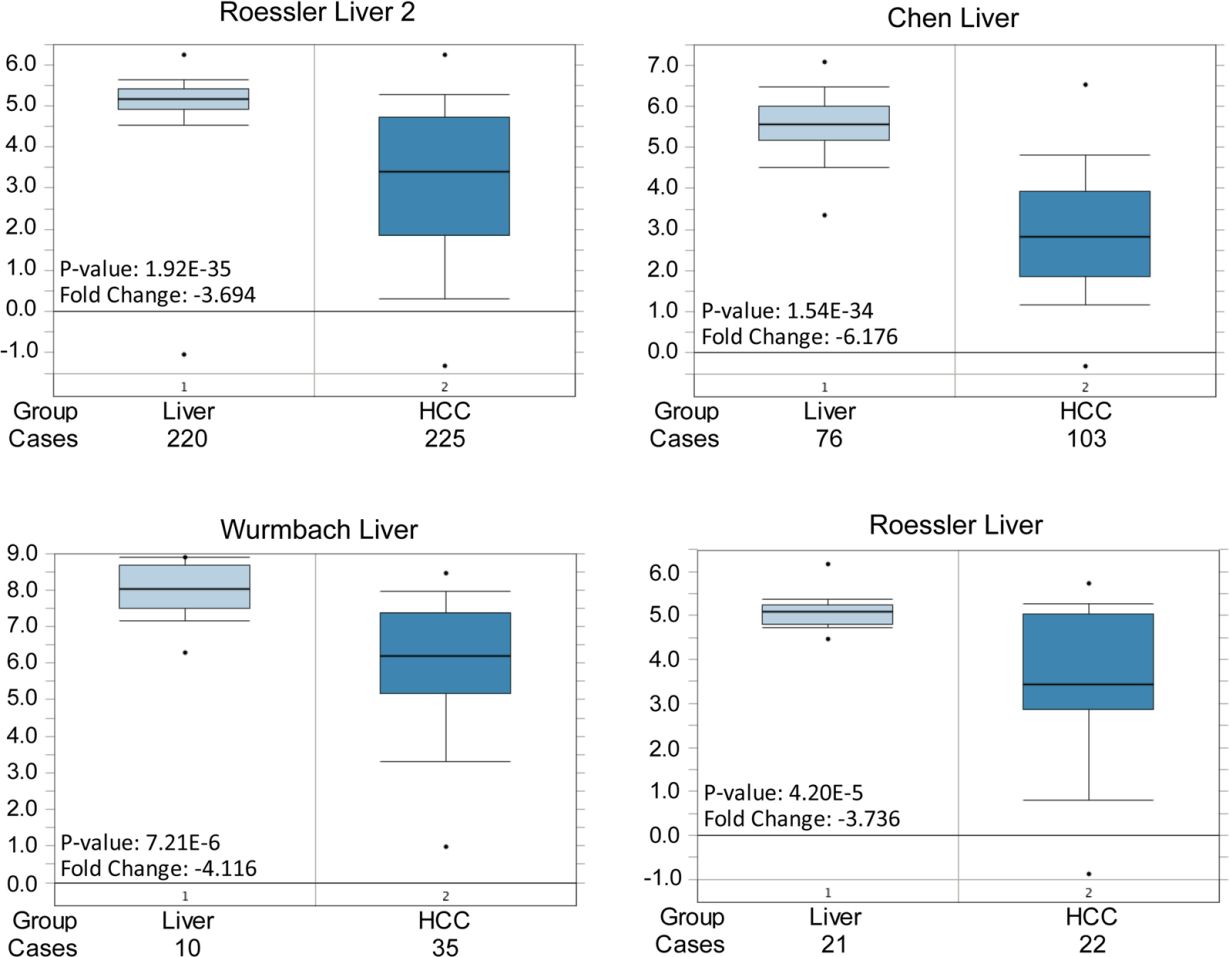
The differential expression of AGXT mRNA between HCC vs. normal liver was analyzed by Oncomine. The expression of AGXT in HCC vs. normal liver were searched and analyzed by Oncomine (https://www.oncomine.org/). The differential AGXT mRNA expression analyses between HCC vs. normal were feasible in four datasets, including: Roessler Liver 2, Chen Liver, Wurmbach Liver and Roessler Liver, totally containing data from 327 livers and 385 HCC tissues. Compared to normal liver, the expression of AGXT mRNA significantly decreased in HCC through all of the four datasets with the fold-changes ranged from − 3.694 to − 6.176, P-values ranged from 1.92E−35 to 4.20E−5

According to the mRNA expression in normal human tissues from GTEx, Illumina, BioGPS, and CGAP SAGE for AGXT gene; as well as protein expression in normal tissues and cell lines from ProteomicsDB, MaxQB, and MOPED for AGXT gene, we found that AGXT was highly expressed in liver, fetal liver and liver secretome (Additional file 4: Figure S1, https://www.genecards.org/). AGXT protein is mostly localized in the peroxisomes where it is involved in glyoxylate detoxification. The expression of AGXT in HCC vs. normal liver were searched and analyzed by Oncomine (https://www.oncomine.org/). The differential AGXT mRNA expression analyses between HCC vs. normal were feasible in four datasets, including: Roessler Liver 2 [61], Chen Liver [62], Wurmbach Liver [63] and Roessler Liver [61], totally containing data from 327 livers and 385 HCC tissues. Compared to normal liver, the expression of AGXT mRNA significantly decreased in HCC in all of the four datasets with the fold-changes ranged from − 3.694 to − 6.176, P-values ranged from 1.92E−35 to 4.20E−5 (Fig. 4). The cohort used for survival risk prediction of the 37 high frequent genes was partially overlapped with Roessler Liver 2 [20, 61].

**Fig. 5:**
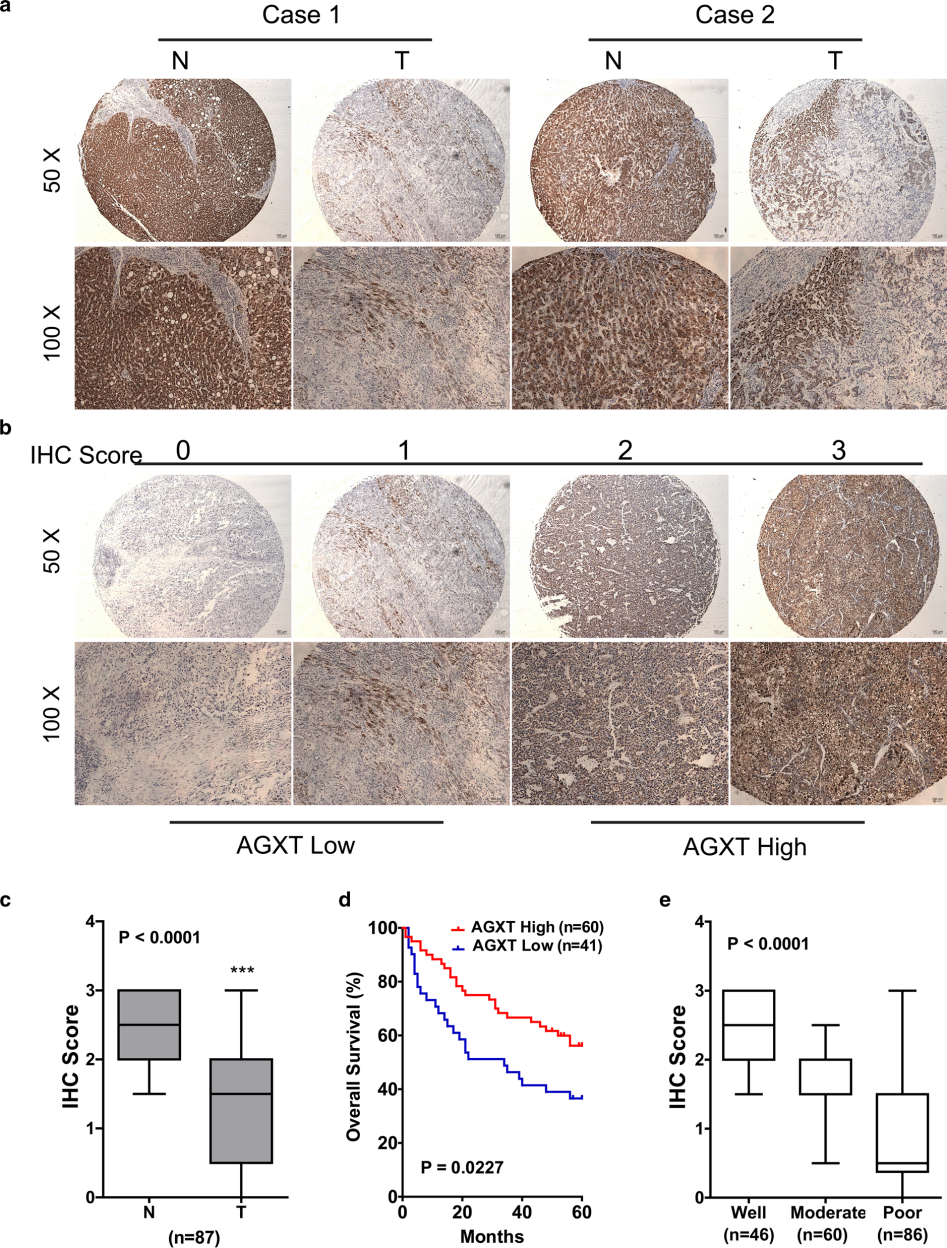
Protein expression of AGXT in HCC and the correlation to prognosis and tumor differentiation. **a** The protein expression of AGXT in paired nontumor (N) and tumor (T) tissues from HCC patients was detected by immunohistochemistry (IHC). **b** Immunostained slides were scored from 0 to 3 according to staining intensity and percentage of positive cells. **c** AGXT IHC scoring of 87 paired HCC samples which contained both nontumor (N) and matched tumor (T) tissues. **d** Kaplan–Meier survival analysis for 101 HCC patients based on AGXT expression, P = 0.0227. **e** The AGXT expression in well, moderate and poor differentiated HCC, ***P < 0.0001

The differential expression of AGXT in liver and HCC was validated by immunohistochemistry (IHC) study with tissue microarray from an independent cohort of 192 HCC patients. Comparison of AGXT by IHC between exactly paired HCC tumor and nontumor tissues was applicable in 87 cases. The IHC staining of AGXT in nontumor liver tissues was strongly positive and was characterized by a granular, diffuse, cytoplasmic staining pattern; while in tumor tissues, the staining of AGXT on malignant hepatocytes were focal and/or pale in a majority of cases (Fig. 5a). Immunostained slides were analyzed with a semi quantitative scoring approach as described in “Methods”, and were scored from 0 to 3 according to the staining intensity (Fig. 5b). The IHC observation reaffirmed that AGXT was highly expressed in nontumor liver tissues and was down-regulated in HCC tumor tissues (P < 0.0001) (Fig. 5c).

### Loss of AGXT expression was correlated with a poor prognosis and differentiation of HCC

As described earlier, under-expression of AGXT correlated to poor prognosis in HCC cohort GSE14520 (Fig. 3), we tried to verify the prognostic value of AGXT in this tissue array HCC cohort. Totally 101 out of the 192 HCC patients that had integrated clinical and follow up data were included, and were divided into two groups based on IHC scores: AGXT high (IHC score 2 and 3, n = 60) and AGXT low (IHC score 0 and 1, n = 41) (Fig. 5b). Overall survival was compared between AGXT high and low cases. It confirmed that low expression of AGXT was associated with poor prognosis (P = 0.0348) (Fig. 5d).

When examining the immunostained HCC slides, we noticed that the staining of AGXT tended to be stronger in well differentiated HCC, but weaker or totally lost in poorly differentiated HCC. Combined with the above finding that AGXT was highly expressed in nontumor liver but down-regulated in HCC, we wondered that if AGXT expression represented the degree of hepatocyte differentiation and maturation. We then compared the IHC score of AGXT among well, moderate and poorly differentiated HCCs. All of the 192 HCC patients were involved. The well differentiated HCCs showed the highest score of AGXT, while the poorly differentiated HCCs scored the lowest. The expression of AGXT in HCC was significantly associated to tumor differentiation (P < 0.0001) (Fig. 5e).

To further explore the clinical parameters associated with AGXT in HCC, we investigated the correlation between AGXT expression and clinicopathological characteristics by performing univariate Cox proportional hazards regression analyses. The 101 HCC patients that had integrated clinical data were included. We found that AGXT expression levels only related to tumor differentiation (P < 0.0001), and there was no significant correlation with patient’s age, gender, serum AFP, tumor size, TNM stage, tumor number, vascular invasion, portal vein tumor thrombus and liver cirrhosis (Table 2). The above observations suggested that reduced expression of AGXT related to tumor differentiation and predicted poor prognosis in HCC.

**Table 2:** Correlation between AGXT expression and clinicopathological features in 101 HCC patients

Variable	AGXT		P-value
	Low (n = 41)	High and moderate (n = 60)	
Age			0.277
≤ 50	16	30	
> 50	25	30	
Gender			0.257
Male	28	47	
Female	13	13	
HBsAg			0.092
Positive	34	56	
Negative	6	3	
Portal vein tumor thrombus			0.930
No	17	38	
Yes	1	2	
Cirrhosis			0.499
Yes	28	40	
No	8	16	
Tumor number			0.882
Single	13	31	
Multiple	3	8	
Serum AFP (ng/ml)			0.078
≤ 300	15	32	
> 300	17	16	
Vascular invasion			0.984
Yes	17	25	
No	24	35	
Tumour size (cm)			0.955
≤ 3	19	27	
> 3	22	32	
Tumor differentiation			< 0.0001*
Well	0	20	
Moderate	7	32	
Poor	34	8	
TNM			0.720
I	18	25	
II	17	24	
III–IV	3	6	

*Significant P values are highlighted in italic

### Loss of AGXT expression promoted the malignant phenotypes of HCC cell lines

**Fig. 6:**
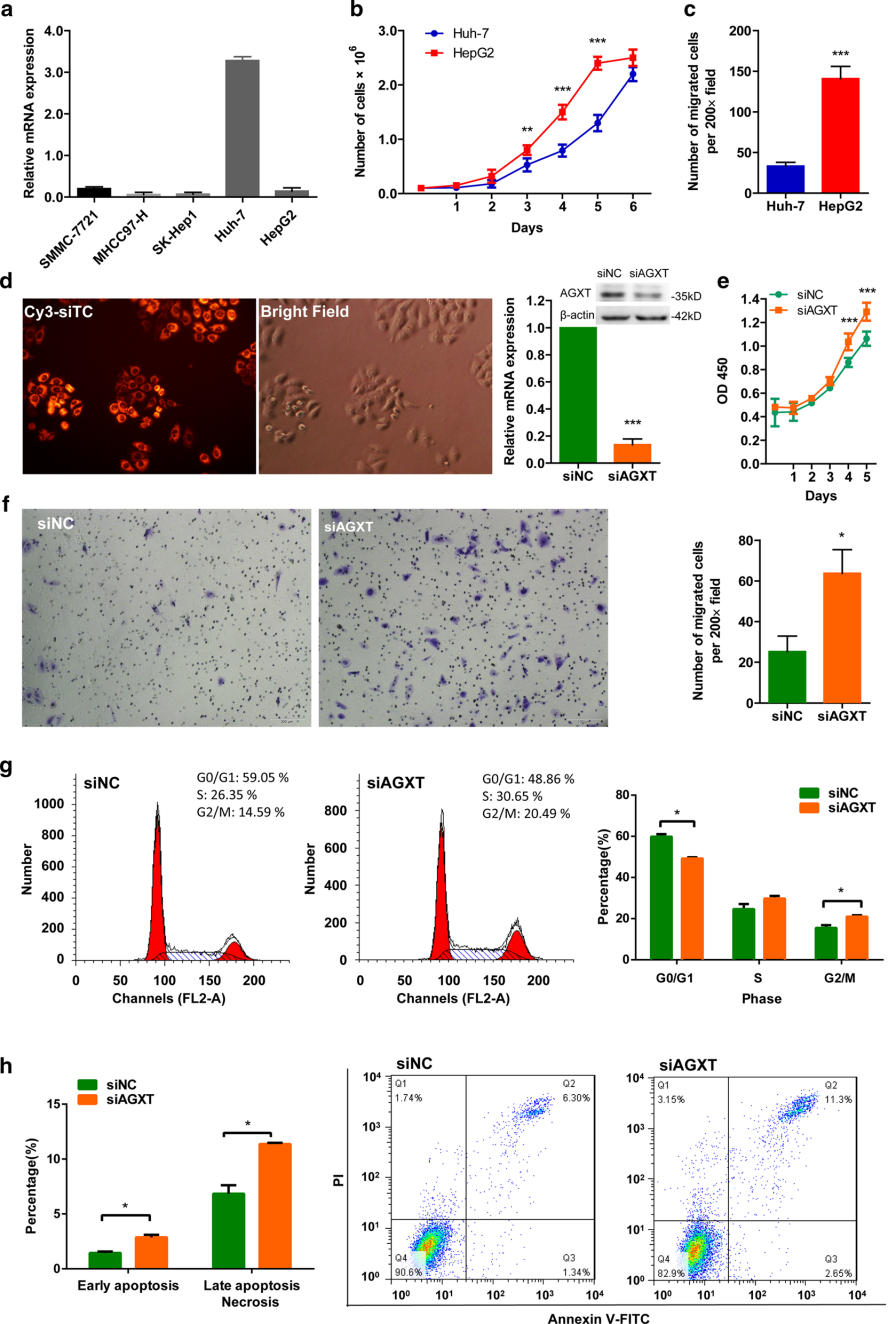
Loss of AGXT expression promoted the malignant phenotypes of HCC cell in vitro. **a** The mRNA expressions of AGXT in a panel of HCC cell lines were detected by quantitative real-time PCR. **b** Proliferation ability of HCC cells was measured by cell counting. **c** Transwell migration assay for HCC cells. **d** The transfection efficiency of siRNA was measured 48 h after Cy3-siTC transfection (original magnification, ×400); and the knockdown efficiency of siAGXT in Huh7 cells was determined by quantitative real-time PCR and western blot. **e** Proliferation of siAGXT transfected Huh7 cells was measured by CCK8 assay. **f** Transwell migration assay for siAGXT transfected Huh7 cells, representative images and quantitative results were provided (original magnification, ×200). **g** Cell cycle assay for siAGXT transfected Huh7 cells, representative histogram of the gated cells in G0/G1, S, and G2/M phases and quantitative analyses, at least 10,000 cells per sample. **h** Apoptosis assay, representative histogram and quantitative analyses of the cells in each phase, at least 10,000 cells per sample. Each bar represents mean ± SD of the data obtained from three independent experiments. ***P < 0.0001, **P < 0.001, *P < 0.05

To further understand the role of AGXT in HCC progression, the biofunctions of AGXT was investigated in human HCC cell lines. First, we compared the expression of AGXT in a panel of HCC cell lines by quantitative real-time PCR. It turned out that Huh-7 exhibited very high level of AGXT, whereas others, such as HepG2, SMMC-7721, MHCC97-H and SK-Hep1 expressed little AGXT (Fig. 6a). Compared to AGXT high-expression Huh-7, AGXT low-expression HepG2 showed higher proliferation ability (P < 0.0001) and greater migration activity (P < 0.0001) (Fig. 6b, c). To further verify the effects of reduced AGXT expression on HCC cells, AGXT high-expression Huh-7 cells were transfected with siAGXT. The transfection efficiency exceeded 95% as it was evaluated by Cy3-siTC. The expression of AGXT was effectively down-regulated by siAGXT at both mRNA and protein levels as validated by RT-PCR and western blot analyse*s* (Fig. 6d). By CCK8 proliferation assay and transwell migration assay, we verified that reduced AGXT expression in Huh-7 accelerated cell proliferation (P < 0.0001) and promoted cell migration (P < 0.0001) (Fig. 6e, f).

The potential effects of AGXT on cell cycle and apoptosis in HCC cells were further examined by flow cytometry. We found that siAGXT treated HCC cells showed a cell cycle shift from G0/G1 to S and G2/M phases (Fig. 6g) indicating enhanced proliferating activity, which was consistent with the CCK8 proliferation assays mentioned earlier. In apoptosis assays, early apoptotic cells were Annexin V-FITC (+) PI (−) (low right quadrant) and late apoptotic/necrotic cells were Annexin V-FITC (+) PI (+) (upper right quadrant). The proportions of both early apoptotic and late apoptotic/necrotic cells increased as the expression of AGXT decreased (Fig. 6h). Increased apoptosis/necrosis along with increased proliferation suggested the controls that integrate cell proliferation and death in normal tissues persist during malignancy. Proapoptotic factors can induce proliferation of neighboring surviving cells to replace dying cells [64, 65]. Increased apoptosis/necrosis along with increased proliferation are always associated with malignant tumors, and are more noticeable in high grade tumors [64, 66].

## Discussion

To date, a large proportion of existed systematic reviews or meta studies on HCC markers were about serum markers [67, 68], and others were focused on one or a set of genes [69, 70], inflammatory-based markers [71, 72]. Due to the differences across experimental methods, sample size and quality, inconsistent annotation and the methods used for data processing and analysis, it is difficult to do integrated bioinformatics analysis with microarray data from different platforms, and sometimes even the raw data are not available in public databases, so there are very few studies that combined the data form different HCC gene expression profiles [73, 74]. Zhang et al. reanalyzed three publicly available datasets of gene expression profiles in the Oncomine database; they identified 17 hub genes (10 unregulated and 7 down-regulated in HCC tissues). Among the 17 genes, 13 genes (SMAD2, PTK2, MAPK1, HDAC1, CDC25A, IGFI, FOS, ESR1, EGFR, SOCS3, SP1, YY1 and JUNB) have been identified as an HCC-related gene [73]. Shi et al. analyzed the integrated microarray data from four independent studies (GSE14520, GSE25097, GSE36376 and GSE57957) from public databases Gene Expression Omnibus (GEO, http://www.ncbi.nlm.nih.gov/geo); they found that KLHL21 was a potential target for therapeutic intervention [74].

In the present study, we conducted a systematic review of studies that reported cDNA microarray data and differentially expressed genes between HCC tumor and nontumor tissues. We searched the PubMed databases for eligible studies published in English-language before July 2016 and retrieved 392 articles. The list of differentially expressed genes from 43 carefully designed studies that satisfied further inclusion criteria were summarized. A total of 1917 HCC patients were involved and 2022 non redundant abnormally expressed genes in HCC were extracted. The frequencies of reported genes were ranked. We finally obtained a list of only five genes (AGXT; ALDOB; CYP2E1; IGFBP3; TOP2A) that were differentially expressed in tumor and nontumor tissues across studies, and were significantly correlated to HCC prognosis. Among the five genes, four (ALDOB; CYP2E1; IGFBP3; TOP2A) were well documented HCC related genes, while AGXT was mostly studies in primary hyperoxaluria type 1 [57, 58], but had not been reported in HCC.

Unlike the unstable changes of the other four genes, the expression of AGXT was constantly reduced in tumors compared to nontumor tissues in a variety of malignant, including liver cancer, gastric cancer, kidney cancer, lung cancer and pancreatic cancer (Additional file 3: Table S3). In the present study, we found that the expression of AGXT reflected the differentiation of HCC and reduced AGXT expression was correlated to poor overall survival of HCC patients. Knocking down of AGXT in HCC cell line could induce a cell cycle shift from G0/G1 to S and G2/M together with enhanced cell proliferation, increased cell death and migration, suggesting a role of AGXT in promoting tumor progression. We provided a high level of evidence on AGXT to serve as a new biomarker and prognostic factor that related to tumor differentiation and progression for HCC.

AGXT gene encoded an enzyme called alanine-glyoxylate and serine-pyruvate aminotransferase which is found in liver cells, specifically within peroxisomes. Serine-pyruvate aminotransferase converts glyoxylate to glycine and is involved in glyoxylate detoxification. The role of AGXT in tumor biology has not been reported yet. One relevant observation is that the metabolic changes to pyruvate and glyoxylate might contribute to the improved therapeutic effects of sorafenib and everolimus combination therapy for HCC [75]. The underlying mechanisms for the contribution of decreased AGXT expression to HCC progression deserved further investigation.

As we were preparing the manuscript, another study group reported AGXT as a novel immunohistochemical marker for the diagnosis of HCC, and demonstrated comparable specificity and higher sensitivity of AGXT compared to arginase-1 [76]. The reason why they were interested in AGXT was uninterpreted; they only mentioned that AGXT was expressed in the liver exclusively. Their finding reaffirmed that AGXT as a new HCC biomarker, and backed up the feasibility and necessity of systematic review on discovering new and reliable biomarkers for HCC as well as for other cancer.

## Conclusion

In conclusion, the present study was an effort to merge abnormally expressed genes in hepatocellular carcinoma form multiple independent studies, which increased sample size and enhanced reliability. The data of present study identified a list of only five high frequency prognostic genes and emphasized a critical role of AGXT deletion during HCC progression, indicated a potential relevance of AGXT restoration for HCC therapy. Further well-designed and larger sample studies are surely warranted to identify the role of the AGXT in the development and progression of HCC and other malignant tumors.

## Supplementary information

**Additional file 1: Table S1.** Clinical characteristics of patients in HCC tissue array cohort at the time of surgery (n = 101).

**Additional file 2: Table S2.** Frequency of the abnormally expressed genes in HCC.

**Additional file 3: Table S3.** The publication history and the expression of the 37 high frequency genes in liver cancer and other malignant tumors.

**Additional file 4: Figure S1.** Expression for AGXT Gene. (A) mRNA expression in normal human tissues from GTEx, Illumina, BioGPS, and CGAP SAGE for AGXT Gene. (B) Integrated Proteomics: protein expression in normal tissues and cells from ProteomicsDB, MaxQB, and MOPED for AGXT Gene. (https://www.genecards.org/).

## Data Availability

All data included in the present study were presented in the main manuscript.

## Abbreviations

AGXT: alanine-glyoxylate and serine-pyruvate aminotransferase
ALDOB: aldolase B
CYP2E1: cytochrome P450 family 2 subfamily E member 1
HBV: hepatitis B virus
HCC: hepatocellular carcinoma
HCV: hepatitis C virus
IHC: immunohistochemistry
IGFBP3: insulin-like growth factor binding protein-3
OS: overall survival
TMAs: tissue microarrays
TOP2A: topoisomerase 2α
