# Supplementary material for: Loss of alanine-glyoxylate and serine-pyruvate aminotransferase expression accelerated the progression of hepatocellular carcinoma and predicted poor prognosis
Source: J Transl Med. 2019 Nov 26;17:390. doi: 10.1186/s12967-019-02138-5 (PMC6880547; doi:10.1186/s12967-019-02138-5)
Supplement: Supplementary file 4 — Additional file 4: Figure S1. Expression for AGXT Gene. (A) mRNA expression in normal human tissues from GTEx, Illumina, BioGPS, and CGAP SAGE for AGXT Gene. (B) Integrated Proteomics: protein expression in normal tissues and cells from ProteomicsDB, MaxQB, and MOPED for AGXT Gene. (https://www.genecards.org/). [file 12967_2019_2138_MOESM4_ESM.doc]

Figure S1.

A
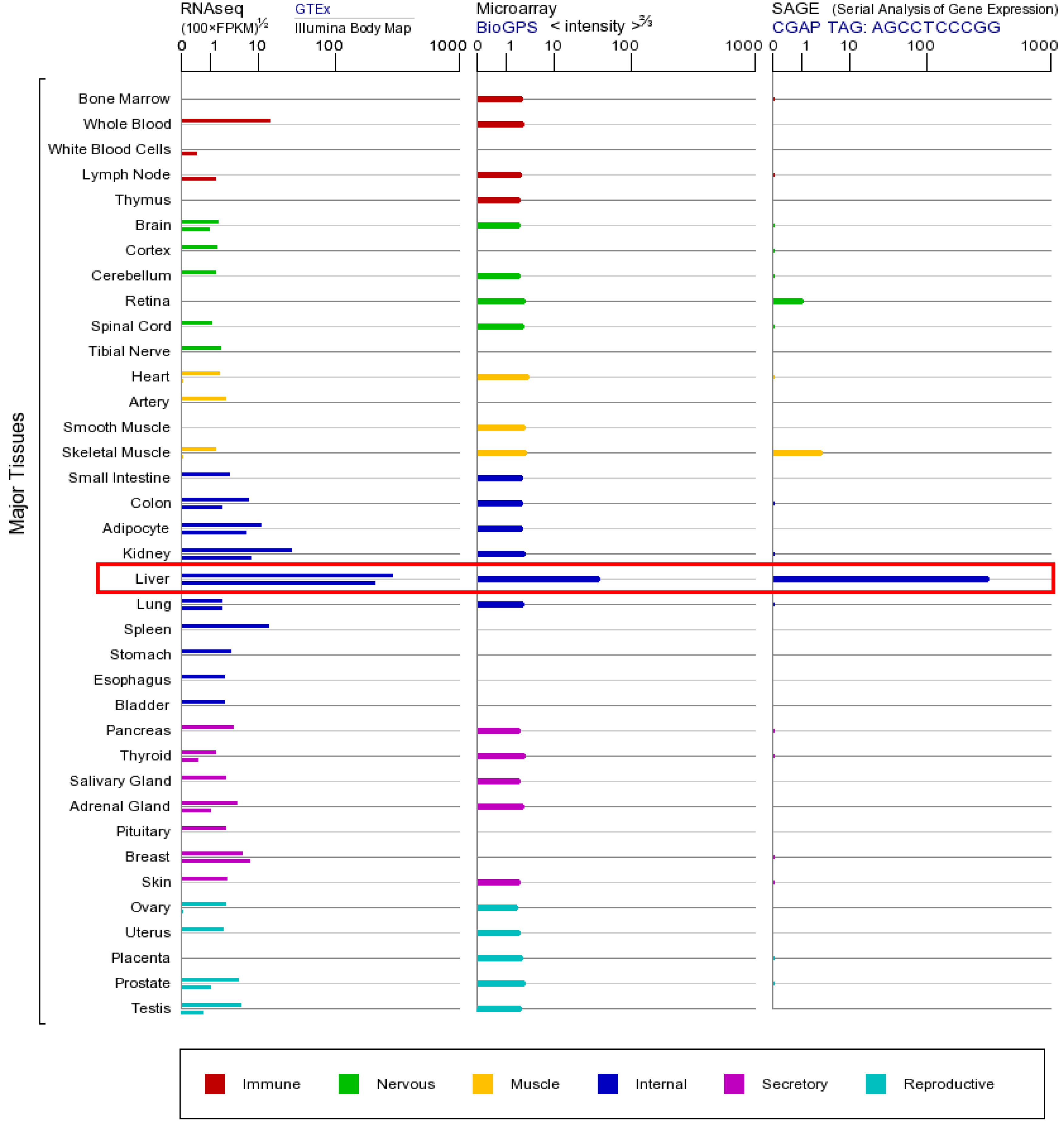


B
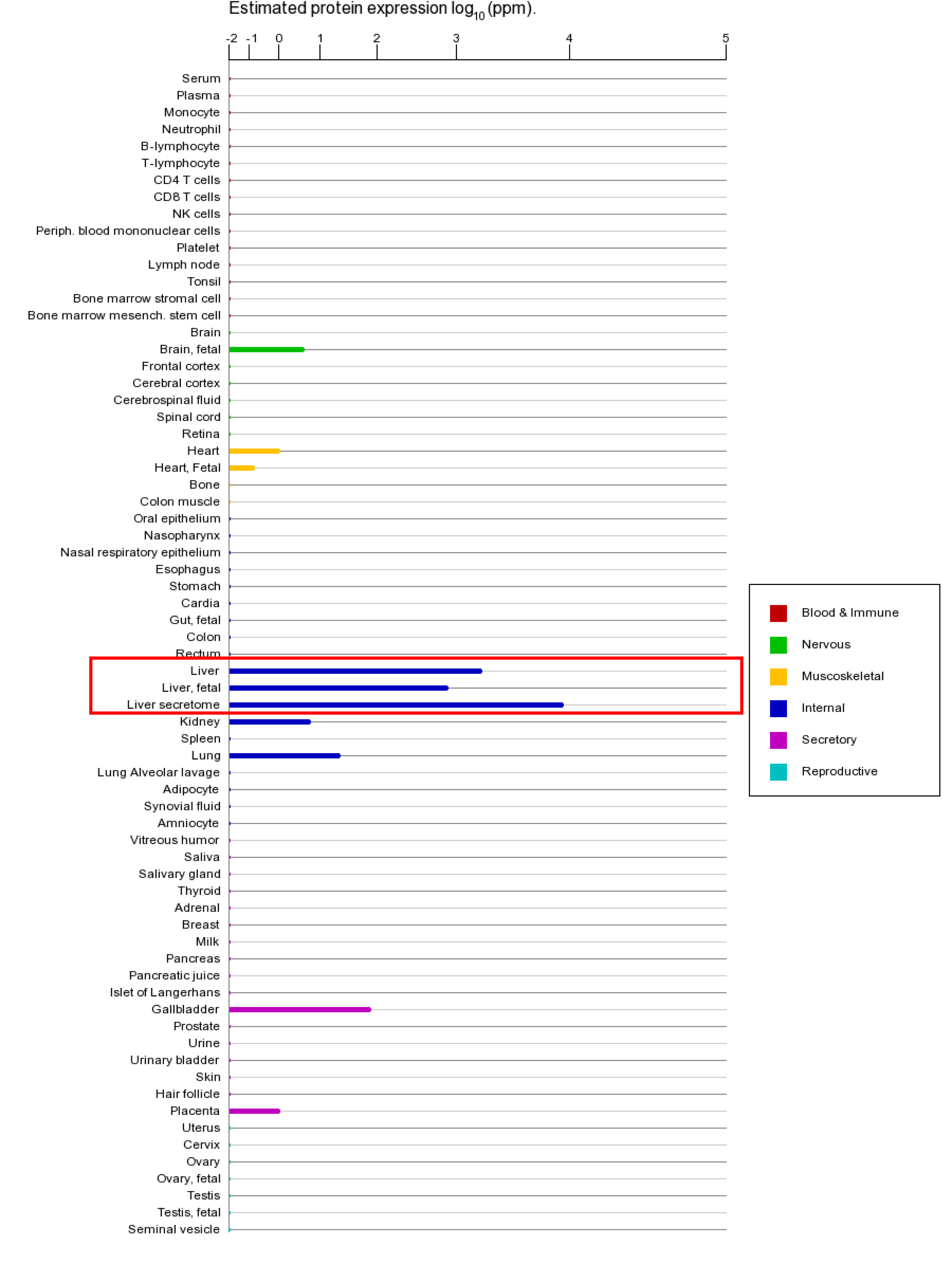


Figure S1. Expression for AGXT Gene. (A) mRNA expression in normal human tissues from GTEx, Illumina, BioGPS, and CGAP SAGE for AGXT Gene. (B) Integrated Proteomics: protein expression in normal tissues and cells from ProteomicsDB, MaxQB, and MOPED for AGXT Gene. (<https://www.genecards.org/>)
